# A bibliometric exploration of affective factors in mathematics education: research trends, thematic evolution, and topic modeling

**DOI:** 10.3389/fpsyg.2025.1746934

**Published:** 2026-01-21

**Authors:** Mehtap Taştepe, Abdulkadir Özkaya

**Affiliations:** 1 Department of Mathematics Education, Faculty of Education, Sinop University, Sinop, Türkiye; 2 Department of Science Education, Faculty of Education, Mustafa Kemal University, Hatay, Türkiye

**Keywords:** affective factors, mathematics anxiety, mathematics education, teacher beliefs, technology integration, topic modeling, trend topic analysis

## Abstract

This study aims to examine research trends in the field of affective factors in mathematics education between 1978 and 2025 using trend topic analysis. A total of 2,587 articles systematically collected from the Web of Science and Scopus databases were analyzed using topic modeling, hierarchical clustering, and multidimensional scaling techniques. The findings reveal that the volume of research in the field has steadily increased, but there has been a shift in thematic focus from broad topics to more specific and contextual areas. While “math anxiety” emerged as the most dominant research focus, teachers’ affective characteristics (Topic 4) and math anxiety (Topic 6) were the fastest growing themes in recent years. In contrast, the intersection of technology and materials with affective factors (Topic 1) and positive psychology-focused concepts (enjoyment, curiosity, flow) have been insufficiently researched. Hierarchical clustering showed that the topics fell into two main clusters: teaching/achievement-focused (Topics 2, 4, 5, 6) and context-factor-focused (Topics 1, 3, 7). The study highlights the asymmetric distribution and research gaps in the field, suggesting that future studies should focus on areas such as positive psychology, integrated theoretical models, and technology-based interventions. Furthermore, the importance of teacher training and math anxiety intervention programs is emphasized.

## 1 Introduction

Mathematics is a fundamental discipline that determines individuals’ success in their academic and professional lives, spanning a wide range from abstract thinking to solving everyday problems. However, this discipline is also at the center of intense anxiety, lack of self-confidence, and negative attitudes for many students worldwide. While traditional mathematics education has focused primarily on cognitive skills and pedagogical methods, research over the past half-century has demonstrated that affective and psychological factors are at least as decisive as cognitive skills (Dowker et al., 2016). Students’ attitudes toward mathematics, their anxieties, motivations, self-efficacy beliefs, and tolerance not only shape their participation in learning processes but also influence their long-term academic success and even their career choices (Jansen and Middleton, 2011; Hannula, 2012).

Within this framework, a growing body of bibliometric research has sought to map trends and intellectual structures in mathematics education. However, most existing bibliometric studies have either examined the field at a general level or focused on limited affective constructs and relatively short time spans (Zawacki-Richter et al., 2020; Donthu et al., 2021). While these studies provide valuable insights into publication growth, collaboration patterns, and dominant themes, they often lack a long-term, psychologically integrated perspective that captures the thematic evolution of affective factors in mathematics education (Donthu et al., 2021). The present study positions itself as an extension and advancement of this literature by systematically synthesizing affective research in mathematics education over an extended temporal window and by integrating educational and psychological perspectives within a unified bibliometric framework.

Accordingly, the importance of this study lies in its holistic examination of this complex structure by bringing together seven distinct thematic focuses that have emerged at the intersection of mathematics education and educational psychology. By adopting a longitudinal and multidimensional bibliometric approach, the study aims to systematically reveal the critical role of affective factors in mathematics achievement and to provide researchers and educators with a comprehensive perspective on the field’s evolution. Furthermore, by examining the interaction between affective dimensions and emerging themes such as technology use and instructional materials, the study seeks to offer insights into contemporary educational challenges. In this context, the findings are expected to inform the design of innovative pedagogical approaches and intervention programs that explicitly incorporate affective processes into mathematics education.

### 1.1 Technology and materials in studies related to affective factors in mathematics education

While it is known that the affective dimension is as decisive as cognitive gains in mathematics education, recent studies show that the integration of technology and materials can have a transformative effect on these affective factors. A review of the literature reveals that technology reduces math anxiety by concretizing abstract concepts (Moyer-Packenham et al., 2019), strengthens self-efficacy (Hung et al., 2014), and increases intrinsic motivation through discovery-based learning (Zydney and Warner, 2016). Similarly, it has been reported that concrete materials and game-based applications positively affect students’ attitudes toward mathematics (Bondurant, 2015) and significantly increase engagement in class (Ke, 2019). However, the literature emphasizes that for these positive effects to occur, technology must be integrated with a pedagogical purpose (Bray and Tangney, 2017) and teacher roles must be adapted to this process.

Recent studies further indicate that artificial intelligence–supported learning environments and adaptive digital tools can enhance students’ emotional engagement and reduce anxiety when aligned with meaningful mathematical tasks (Awang et al., 2025; Dignam et al., 2025).

### 1.2 Instructional and contextual variables shaping affective factors

Students’ feelings toward mathematics are not independent of their learning environment. In this context, constructivist teaching methods such as collaborative learning, problem-based learning (PBL), and flipped classrooms support students’ sense of belonging and need for autonomy by placing them at the center. Collaborative learning environments, in particular, minimize social comparisons, thereby reducing anxiety and strengthening academic resilience (Slavin, 2014). Similarly, instructional designs incorporating gamification elements trigger the need for success, creating deeper psychological engagement in the learning process. Such interventions explore effective ways to positively change affective factors. Recent classroom-based meta-analyses confirm that student-centered instructional designs are associated with sustained improvements in motivation and emotional regulation in mathematics learning contexts (Kohler et al., 2024).

### 1.3 Psychological reflections of demographic differences: stereotype threat and self-efficacy

Demographic variables such as gender, socioeconomic status, and ethnic origin have an of the fundamental psychological mechanisms underlying this effect is “stereotype threat.” For example, individuals exposed to a negative stereotype such as “girls are not good at math” may enter a psychological state where their anxiety levels increase due to the fear of confirming this stereotype, and consequently perform below their normal level (Spencer et al., 1999). This situation can erode self-efficacy beliefs, causing individuals to shy away from higher-level fields related to mathematics and careers such as. This theme focuses on understanding the invisible psychological barriers underlying inequalities. Recent longitudinal studies demonstrate that stereotype threat continues to operate in digital and assessment-driven learning environments, highlighting the need for equity-oriented instructional designs (El Chaabi and Younes, 2025).

### 1.4 The teacher’s psychological profile: the reflection of affective factors in the classroom

Teachers are the most important architects of classroom climate and learning culture. Teachers’ own math anxiety, beliefs about the nature of math (“Is math a fixed ability or can it be developed?”), and teaching self-efficacy directly influence their pedagogical choices and interactions with students. Within the framework of the “mindset” theory, teachers who view mathematics as a “developable ability” (growth mindset) are seen to give their students more supportive feedback and encourage them not to give up in the face of difficulties (Dweck, 2006). Furthermore, experimental studies have shown that teachers’ own math anxiety creates an “emotional contagion” effect, particularly on girls’ achievement and anxiety (Beilock et al., 2010). Therefore, teacher education programs should aim to develop not only candidates’ subject knowledge but also these critical psychological factors (Akin and Kurbanoglu, 2011).

Recent evidence suggests that professional development programs explicitly addressing teachers’ affective profiles lead to measurable improvements in classroom emotional climate and student motivation (Wang et al., 2024).

### 1.5 The causal cycle between affective factors and academic achievement

There is a reciprocal and evolving relationship between affective factors and academic achievement over time. High mathematical self-efficacy and positive attitudes encourage students to exert more effort and tackle challenging problems, thereby increasing success. Achieved success, in turn, further reinforces self-efficacy beliefs and positive attitudes. Conversely, low achievement can create a vicious cycle by feeding anxiety and negative beliefs (Marsh and Craven, 2006). Longitudinal studies show that math anxiety in elementary school can predict math success in high school, independent of cognitive ability (Ma and Xu, 2004). This relationship proves that affective factors are not only a result but also a powerful cause of success. Recent large-scale panel studies confirm this bidirectional relationship and emphasize that affective interventions can alter long-term achievement trajectories (Zhu et al., 2024).

### 1.6 Math anxiety: the clash of cognitive and emotional systems

Math anxiety should be considered a domain-specific anxiety disorder. From a psychological perspective, anxiety blocks working memory, the executor of cognitive processes, via the amygdala, the brain’s emotional processing center (Ashcraft and Krause, 2007). A highly anxious student both attempts to solve problems during an exam and consumes mental resources to suppress negative thoughts such as “what if I fail?” This situation leads to a decrease in current cognitive capacity and poor performance. Therefore, math anxiety is a serious psychopedagogical problem that must be addressed, resulting in learned helplessness and avoidance behavior. Neuroscientific studies published in recent years provide converging evidence that emotion-regulation strategies can mitigate the cognitive load imposed by math anxiety (Martell et al., 2025).

### 1.7 The emotional legacy of the family environment: early psychological transmission

The family is the primary social context where children acquire their initial attitudes and beliefs about mathematics. Parents’ own math anxiety can be unconsciously transferred to their children. In particular, an anxious parent who helps their child with math homework can be more detrimental to the child’s learning than not helping at all (Maloney et al., 2015). Furthermore, the value parents place on mathematics and the use of “mathematical language” at home (talking about numbers and shapes) enables children to see mathematics as a meaningful part of daily life. Therefore, family-based interventions are seen as an early and effective strategy for supporting children’s mathematical development. Recent intervention studies highlight that family-based affective support programs significantly reduce early math anxiety and promote positive beliefs in primary school children (Wang et al., 2025).

In conclusion, understanding mathematics education is not possible without considering its psychological dimension. There is a need for multidisciplinary work across a wide range of areas, from technology to teacher training, family involvement, and the perspective of social justice, which treats the student as a “psychological whole.” This thematic map will contribute to the development of a holistic understanding that centers the human experience in the mathematics learning process for both researchers and practitioners.

### 1.8 Purpose and original value of this study

This article aims to systematically examine the seven thematic areas outlined above, which are closely interrelated, using quantitative text mining methods such as Topic Modeling. The existing domestic and foreign literature generally addresses these factors in pairs or trios. This study aims to reveal current research trends, focal points, and potential gaps in the psychology of mathematics education by evaluating dynamics such as family, anxiety, self-efficacy, attitude, motivation, gender, teacher, and measurement together. Thus, it hopes to contribute to theoretical models in the field and shed light on the development of more comprehensive and integrative intervention strategies for parents, teachers, and education policymakers. For Original Research Articles, Clinical Trial Articles, and Technology Reports, the introduction should be succinct, with no subheadings. For Case Reports, the Introduction should include symptoms at presentation, physical exams, and lab results.

## 2 Method

In this study, topic modeling was used to reveal thematic developments and patterns of academic interest over time on a defined research topic. Such methods allow for a comprehensive and systematic review of research conducted in a specific field. Through topic modeling and trend analysis, it is possible to track developments in the field over time, identify emerging themes, and analyze shifts in research priorities. The Orange software is used for this purpose. This software first classifies the keywords of article collections using machine learning methods (Demšar and Zupan, 2013). In this way, articles are grouped under common topics. This approach also facilitates the identification of research areas that have been insufficiently researched or neglected.

Methodologically, this study is positioned at the intersection of bibliometric analysis, science mapping, and text-mining–based topic modeling. Unlike traditional bibliometric studies that primarily focus on citation counts, co-authorship networks, or keyword frequencies, the present study adopts a longitudinal, topic-oriented approach to examine the thematic evolution of affective factors in mathematics education. By integrating PRISMA-guided systematic data collection with Latent Dirichlet Allocation (LDA) and trend topic analysis, the study moves beyond descriptive bibliometrics and provides an interpretable mapping of how research themes have emerged, diversified, and shifted over time.

### 2.1 Research design

This study is a mixed-methods research aimed at systematically analyzing studies on affective factors in mathematics education. The research was conducted by combining both a qualitative systematic review and quantitative trend and topic modeling analyses. The qualitative component of the study involved content analysis to reveal thematic patterns in the literature, while the quantitative component included statistical evaluation of numerical data, including the distribution of article topics, temporal trends, and momentum metrics. First, the study numerically examined the ratio of keywords and various numerical quantities and trend patterns. It was determined which topics were studied more and which were studied less. Subsequently, content analysis was performed on all articles to reveal gaps in the trends.

The study process was structured according to the PRISMA (Preferred Reporting Items for Systematic Reviews and Meta-Analyses) protocol to ensure transparency and reproducibility in systematic reviews and analyses (Page et al., 2021). Accordingly, this research is not only a descriptive review but also an approach that analyzes developments in the literature at both qualitative and quantitative levels, supported by data mining techniques and topic modeling methods that reveal trends.

### 2.2 Databases and search strategy

The literature review for this research was conducted using two major academic databases to cover national and international studies with high impact values in the field of mathematics education: Scopus and Web of Science. The search process was conducted between June and September 2025.

The keywords used during the search included the following terms: “mathematical self-efficacy,” “mathematics anxiety,” “mathematics motivation,” “mathematics identity,” “mathematics attitudes,” “mathematics beliefs,” “affective-motivational model,” “mathematics resilience,” “mathematics intervention,” “mathematics enjoyment,” “epistemological beliefs about mathematics,” “mathematics self-concept,” “mathematics curiosity,” “mathematics interest,” “mathematics confidence,” “mathematics disposition,” “mathematics value” “task value in mathematics,” “mathematics flow,” “sense of belonging in mathematics,” “mathematics frustration,” “mathematics boredom,” and “mathematics engagement.” Boolean logical operators (“AND,” “OR”) were used to obtain a more comprehensive and valid result.

For topic modeling, the textual corpus consisted of article titles, abstracts, and author-provided keywords. Full texts were not included due to database access limitations and to ensure consistency across sources. Previous topic modeling and bibliometric studies suggest that titles and abstracts sufficiently represent the conceptual focus of articles while minimizing noise introduced by methodological or contextual sections.

### 2.3 Inclusion and exclusion criteria

During the systematic review phase of the study, literature selection was conducted according to predefined inclusion and exclusion criteria to ensure transparency and consistency. The inclusion criteria required that studies be published between 1978 and 2025, a time frame deliberately chosen to capture the historical emergence, conceptual development, and contemporary diversification of affective research in mathematics education. The late 1970s mark a critical period in which affective constructs such as attitudes toward mathematics, mathematics anxiety, and beliefs began to be systematically examined alongside cognitive dimensions. Including studies from 1978 ensures coverage of the earliest empirically grounded research in this domain, while extending the scope to 2025 allows the analysis to reflect current research trends and recent developments. From a bibliometric perspective, this extended temporal window enables the identification of long-term thematic shifts and macro-level trends in the field. Although an earlier text entry indicated 1987, the analysis was initiated from 1978 in accordance with the dataset structure and previous data tables. Additional inclusion criteria required that studies be conducted within the field of mathematics education, focus on affective factors (e.g., attitude, anxiety, motivation, beliefs), be published as peer-reviewed journal articles, and be written in English. Studies were excluded if they focused solely on cognitive processes, lacked empirical data, were conducted outside the field of mathematics education (e.g., engineering, finance, or physics), were published in formats other than journal articles, or constituted duplicate publications with substantially overlapping content.

### 2.4 Study selection process

Within the scope of this systematic review, a comprehensive literature search was conducted in the Web of Science (WoS) and Scopus databases to identify studies on affective factors in mathematics education. The reason for starting the study in 1978 is that only 5 studies were found scattered across the previous years (33 years).

Initial Screening (Identification):

A total of 4,909 + 5,252 = 10,161 articles were identified as a result of the search, with 4,909 studies from the Web of Science database and 5,252 studies from the Scopus database. Following this initial search, 5,168 common studies (duplicate records) were identified in both search systems.

Screening Phase:

1. Removal of Duplicate Records: After removing the 5,168 common studies from the dataset, a total of 4,993 articles remained for analysis.
2. Exclusion of Non-Article Publications: Upon reviewing the remaining studies, 118 were found to be non-articles, consisting of book chapters, conference proceedings, or symposium abstracts. These studies were excluded from the dataset.
3. Exclusion of Studies Without Abstracts: After excluding non-article type studies, 4,875 articles remained. It was observed that 81 of these articles did not have abstracts and were excluded from the analysis on the grounds that they did not provide sufficient information for content evaluation.
4. Elimination of Off-Topic Studies: Upon reviewing the titles and abstracts of the remaining studies, 2,207 articles were determined to be inconsistent with the research focus (affective factors in mathematics education). These off-topic studies were excluded.

**Figure 1:**
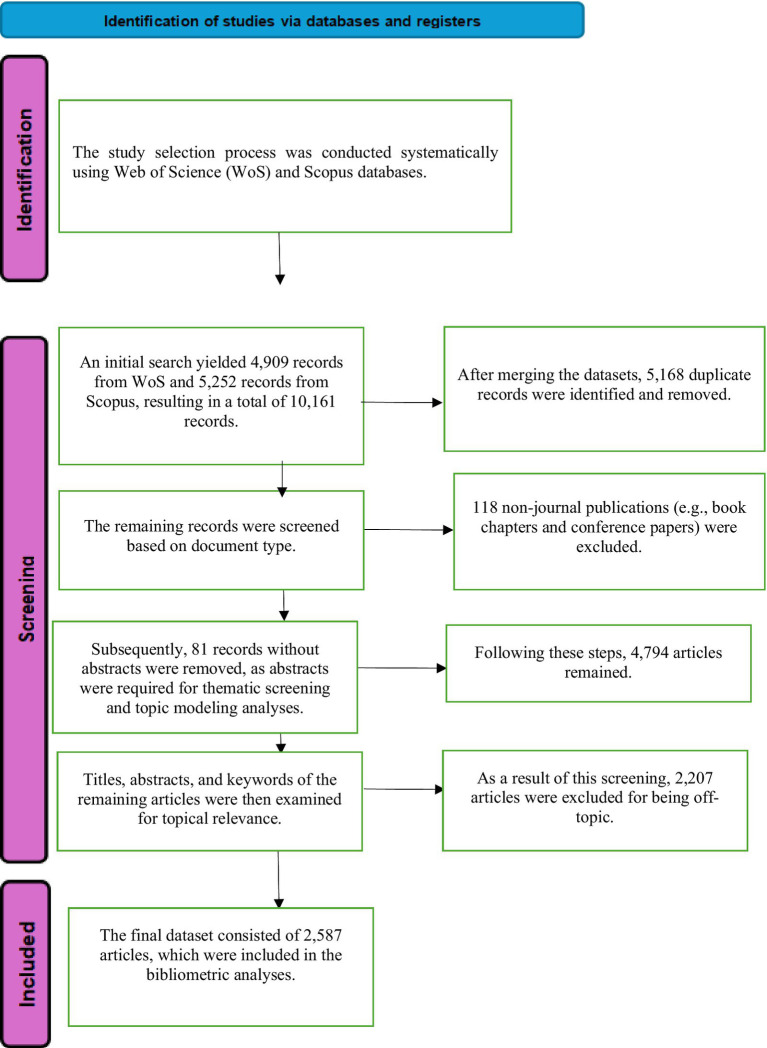
Data selection criteria and screening process.

Final Data Set (Included):

As a result of all the above exclusion processes, a total of 2,587 articles remained to be included in the systematic review. These articles formed the basis of the quantitative and qualitative analyses of the study (Figure 1).

After all these filtering processes, a total of 2,587 articles deemed suitable for systematic analysis were included in the content analysis process. This process was carried out carefully to increase the reliability of the study and clarify the scope of the analysis.

### 2.5 Data coding and analysis method

During the research process, the identified article sets were first divided into thematic groups using machine learning-based topic modeling techniques within the framework of keywords using Orange software. Following this automated classification, a team of two researchers independently examined each thematic group and determined the title and qualitative definition of each topic through qualitative content analysis based on keywords and representative articles.

To ensure inter-coder reliability, the two researchers carried out the qualitative coding process independently during the initial phase. The independently generated topic labels and definitions were then systematically compared, and inter-coder agreement was calculated using percentage agreement. The agreement level exceeded the commonly accepted threshold for qualitative research (approximately 80%), indicating high inter-coder reliability. In cases of disagreement, the researchers conducted iterative consensus meetings to refine topic labels and definitions until full agreement was achieved. The initial agreement level exceeded the commonly accepted threshold in qualitative research, indicating a high degree of consistency between the coders.

In cases where discrepancies occurred, the researchers engaged in iterative consensus meetings, during which disagreements were resolved through in-depth discussion and re-examination of the associated keywords, topic distributions, and sample articles. This process continued until full consensus was achieved for all thematic groups.

After reaching consensus, the finalized thematic structure was reviewed once more to ensure conceptual coherence and consistency with the quantitative outputs generated by Orange software. The content of the defined topics and representative sample articles are presented in Table 1. In addition to this qualitative definition process, Orange software provided rich quantitative information for each topic, including topic prevalence and distribution across the dataset. These quantitative data initially included descriptive information such as the number of articles per topic and the fundamental changes in topics over time. At a more advanced level, this information was presented with detailed metrics such as acceleration values, changes in topic prevalence across different time periods, temporal trends, and volumetric changes. These findings indicate a significant transformation in the thematic focus of the field under study. However, changes in the mere annual number of articles are not, by themselves, a sufficient indicator for determining topic trends. The proportion of topics within the total publication volume and the dynamics of this proportion’s change over time provide more in-depth and contextual information about thematic evolution. Therefore, calculating each topic’s share of the total number of publications in five-year periods is methodologically critical. The use of five-year intervals to analyze the proportional distribution of research topics over time is a well-established methodological approach in bibliometric and science mapping studies. Five-year periods provide a balanced temporal resolution that allows researchers to capture meaningful thematic changes while minimizing short-term fluctuations that may arise from publication delays, database indexing practices, or temporary research trends. Shorter intervals (e.g., annual or biennial analyses) often result in unstable patterns, particularly for emerging or less dominant topics, and may overemphasize temporary variations rather than long-term thematic evolution. In contrast, longer intervals (e.g., 10 years or more) tend to obscure critical transitional phases and delay the detection of shifts in research focus. Therefore, five-year periods represent an optimal compromise between sensitivity to change and analytical stability. This approach is widely adopted in bibliometric research examining thematic evolution, trend dynamics, and intellectual structures across disciplines. Prior studies have demonstrated that five-year segmentation enables clearer interpretation of longitudinal trends and facilitates meaningful comparisons across time periods (e.g., Callon et al., 1991; Cobo et al., 2011; Aria and Cuccurullo, 2017). Accordingly, dividing the study period into consecutive five-year intervals allows for a more robust and interpretable analysis of how the relative prominence of research topics evolves over time within the overall publication landscape. These ratios allow for the evaluation not only of the absolute number of articles on a topic but also of its relative weight within the total publications. For example, despite an increase in the annual number of articles on a topic, a decrease in its proportion within the total publications may indicate that the academic community’s interest has generally shifted to different themes and that the relative importance of that topic has diminished. This approach makes it possible to analyze the volumetric changes and diverging behaviors of topics over time in greater detail.


$$\text{Percentage Rate}=\frac{\text{Number of articles}\;\mathrm{on}\;\text{the topic in the period}}{\text{Total number of articles in the petiole}}\times 100$$

**Table 1:** Topic titles, article examples, and definition.

Topic heading	Sample article	Definition
Topic 1 – Technology and Materials in Studies Related to Affective Factors in Mathematics Education	Ewing (2016a,b), Gu et al. (2011)	This heading covers research emphasizing the central role of technology in understanding and developing students’ affective characteristics (attitude, anxiety, motivation) in the mathematics learning process. The studies examine how digital technologies, learning materials, and pedagogical tools shape students’ emotional experiences, the ways in which these tools are applied, their effects, and their effectiveness. The analysis brings together experimental and situational research in which technology-based interventions are used to support student emotions. The use of technology in data collection and analysis processes is also addressed in this context. In this respect, technology is central to these studies.
Topic 2 – Factors Affecting Emotional Factors in Mathematics Education-Teaching	Utari et al. (2019), En-nhiri et al. (2025), Manaud et al. (2024)	Topic 2 These studies analyze current trends and intervention approaches in this field by examining the *modifying* or *relational* effects of teaching methods (e.g., Collaborative Learning, SBL) and technological tools (e.g., Dynamic Software) on affective variables. The aim is to identify factors that positively influence students’ emotions and attitudes towards mathematics. However, teaching is at the center of this topic.
Topic 3 – The relationship between demographic variables and affective factors in mathematics education	Gao et al. (2025), Nosek et al. (2011), Shapiro et al. (2012)	Topic 3 This heading covers studies that examine the interaction and role of demographic variables such as gender and racial/ethnic origin on students’ affective domains (self-efficacy, interest, motivation, attitude) in mathematics education. Articles specifically investigate how stereotype threat and social cognitive mechanisms mediate the mathematics performance and ultimate STEM career choices of students from different demographic groups. This cluster focuses on the psychological and sociocultural pathways underlying inequalities and achievement gaps in mathematics education.
Topic 4 – The relationship between affective factors and mathematics instruction and teachers and teacher candidates	Cheng and Zhenyi (2025), Shalem et al. (2018), Frommelt et al. (2023)	Topic 4 This heading brings together research examining the development of teachers’ and teacher candidates’ affective characteristics, such as their beliefs about mathematics, self-efficacy, and anxiety levels, and their role in mathematics teaching. The studies address how instructional factors affect teaching quality, student interest, and achievement, as well as how teacher education programs shape these affective states over time. In short, this topic provides an in-depth analysis of the affective dimension of mathematics education from the perspective of classroom practitioners (teachers).
Topic 5 – The impact of affective factors in mathematics education on student achievement and learning	Martin and Debus (1998), Gulburnu and Yildirim (2021), Parker et al. (2015).	Topic 5 This topic covers studies that examine the complex relationships between students’ affective factors, such as their mathematical self-concept, attitudes, beliefs, and motivation, and their academic achievement and career goals. These studies also examine the relationship between conceptual development and affective development. Research analyzes how these affective characteristics function as strong predictors of success and conceptual development, particularly how this relationship is shaped through social comparison mechanisms such as internal and external reference frames. This cluster focuses on methodological and theoretical studies that test the reciprocal relationships between affective and achievement variables and cross-cultural validity. The focus of this topic is on students.
Topic 6 – Anxiety in mathematics education and teaching	Demedts et al. (2022), Núñez-Peña and Campos-Rodríguez (2024), Sokolowski et al. (2019)	Topic 6 This main topic examines the profound and negative role of Math Anxiety (MA) in education and learning processes. It emphasizes that MA is a domain-specific obstacle that reduces students’ math achievement and consumes cognitive resources such as working memory. Therefore, this topic reveals that MA is an undesirable and manageable condition in math education.
Topic 7 – The influence of family on affective factors in mathematics education	Cheung et al. (2025), Eason et al. (2021), Yang et al. (2025)	Topic 7 This main topic examines the effect of parents’ affective factors, such as their own math anxiety, beliefs, and attitudes, on children’s early numeracy skills and interest in mathematics. Research reveals how parents’ negative attitudes shape the home mathematics environment (mathematics-related activities and language use) and, indirectly, their children’s achievement. In particular, parental anxiety and the form of mathematics support (controlling or autonomy-supportive) are shown to be critical mediators for children’s mathematics achievement and anxiety.

While the quantitative data presented earlier provides a preliminary indicator for identifying thematic trends, it falls short of revealing the contextual depth and theoretical orientation within the research field. Therefore, in addition to numerical analysis, an in-depth examination of the articles was deemed necessary.

To this end, the articles under each thematic group (topic) were subjected to qualitative content analysis to be analyzed in detail in terms of content and context. In this particular study, the focus of the analysis was to determine the use and level of examination of variables that constitute affective factors (e.g., attitude, anxiety, motivation, etc.) in research articles, especially in the field of mathematics education. Furthermore, using common content analysis methods, the main objectives of the studies examined were determined, and the researchers systematically identified gaps in the existing literature. This qualitative deepening aimed to go beyond quantitative data and comprehensively reveal the current state of the research field at both the structural and content levels.

### 2.6 Topic modeling and defining the ideal number of topics

In this study, a systematic topic modeling process was followed to reveal research trends in the field of affective factors in mathematics education.

This study analyzed 2,587 articles identified in accordance with the PRISMA protocol from the Web of Science and Scopus databases. First, the texts underwent a preprocessing stage. During preprocessing, all texts were converted to lowercase, and punctuation, numerical characters, and non-alphabetic symbols were removed.

Standard English stopwords were eliminated using the default stopword list provided by the Orange text mining library. Stemming was applied to reduce inflected word forms to their root structures, ensuring semantic consistency across documents. Additionally, extremely low-frequency terms were excluded to reduce sparsity and noise in the document–term matrix. At this stage, all documents were converted to lowercase, stopwords were removed, and stemming operations were applied to make them suitable for analysis.

Then, topic modeling was performed using the Latent Dirichlet Allocation (LDA) algorithm on the text data vectorized using the TF-IDF (Term Frequency–Inverse Document Frequency) method. LDA is a machine learning method that assumes each document can contain multiple topics and that each topic has a distribution represented by specific sets of words.

The LDA model was implemented using the Orange Data Mining platform with text vectorization based on the TF–IDF weighting scheme. Model parameters were set to default symmetric Dirichlet priors for the document–topic (*α*) and topic–word (*β*) distributions, as recommended for exploratory topic modeling in large-scale bibliometric datasets. Multiple iterations were conducted to ensure model stability and convergence.

**Figure 2:**
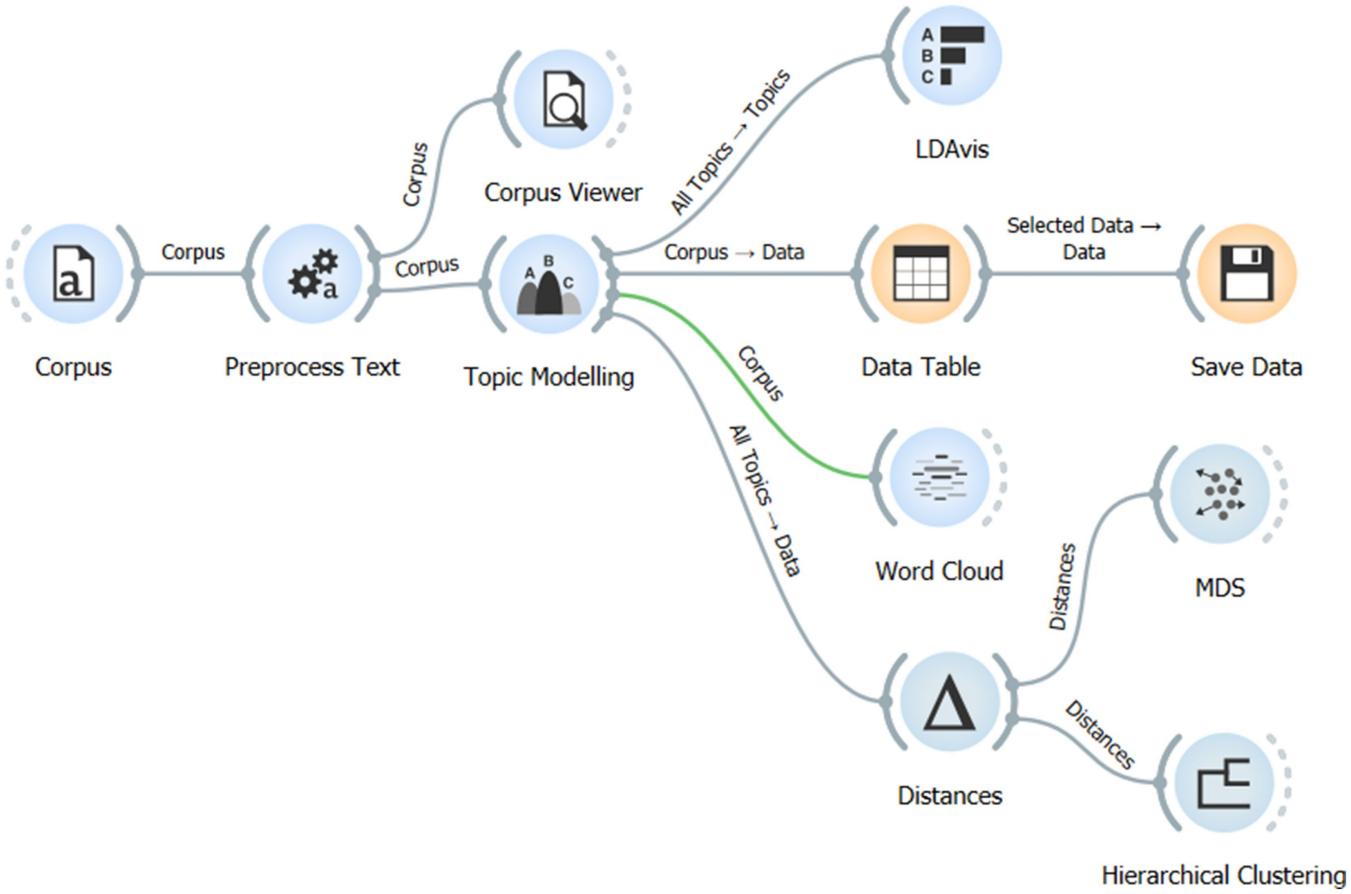
The steps of text preprocessing and topic modeling analysis.

The analysis process was performed using the Orange Data Mining platform (Demšar and Zupan, 2013). This platform has a workflow structure that automates and visually represents the text mining process (Figure 2). The model’s validity and interpretability were assessed using topic coherence values and LDAvis-based visualizations. These visualizations clearly revealed topic distributions and inter-topic relationships, enabling the identification of seven meaningful thematic clusters.

In the final stage, the temporal evolution of these thematic clusters was examined using hierarchical clustering and multidimensional scaling (MDS) techniques, systematically revealing changes in research paradigms in the field.

**Figure 3:**
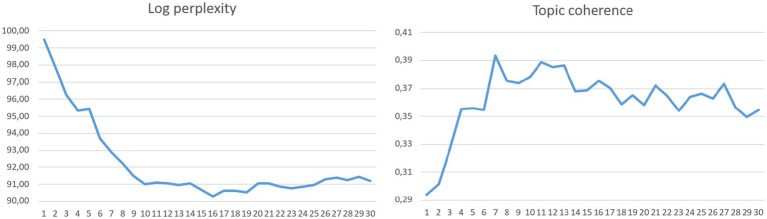
Log perplexity and topic coherence scores across different numbers of topics in the topic modeling process.

In this study, a systematic topic modeling process was followed to reveal research trends in the field of affective factors in mathematics education. Before beginning the topic modeling analysis, determining the ideal number of topics (K) that best represents the thematic structure in the literature is of critical importance. To this end, the K value for the Latent Dirichlet Allocation (LDA) algorithm was increased from 1 to 30, and metrics reflecting the statistical fit of the model for each K value (log-perplexity) and topic coherence (C_v) indicating semantic consistency were calculated. The main objective was to determine the most meaningful K value where high topic coherence and low log-perplexity values intersect. The analysis concluded that seven (7) topics provided the model’s most statistically consistent and semantically interpretable structure. The seven-topic model, presenting 92.89182 log-complexity and 0.39344 topic coherence values, was determined to be the most suitable model for mapping the research data thematically. These seven thematic clusters were defined to present a meaningful and interpretable structure (Figure 3).

## 3 Results

First, when considering the data obtained on search terms, the thematic distribution of research in the field of psychology in mathematics education provides important clues about the current state of the field and future directions. Frequency analyses reveal that the studies exhibit a largely asymmetric distribution. The term “mathematics anxiety” (1197) having by far the highest frequency indicates that the field is largely oriented toward a problem-focused paradigm. This suggests that mathematics anxiety is perceived as a critical psychological barrier for both students and educators and has established a strong research tradition at the intersection of clinical psychology and educational psychology. Structures with medium frequencies, such as “mathematics self-concept” (207), “mathematical self-efficacy” (137), and “mathematics motivation” (134) indicate that social cognitive theory and self-theories hold an important place in mathematics education, and that individuals’ cognitive representations and motivational processes related to mathematics remain on the research agenda.

However, low-frequency terms reveal significant gaps in the field. In particular, concepts centered on positive psychology, such as “mathematics enjoyment” (15), “mathematics curiosity” (2), and “mathematics flow” (0), appear to have been studied quite limitedly. This situation shows that research has largely focused on negative emotions and cognitive processes, while positive emotional experiences and the enjoyment of learning have not been sufficiently investigated. Similarly, the low frequency of social psychological factors such as “sense of belonging in mathematics” (7) indicates that the social and cultural dimensions of mathematics learning have been neglected. Furthermore, the lack of metacognitive processes such as “epistemological beliefs about mathematics” (8) and integrated theoretical frameworks such as the “affective-motivational model” (0) suggests that the field exhibits a fragmented and scattered structure.

The limited number of intervention and application-focused studies is also noteworthy. The low frequency of terms such as “mathematics intervention” (55) and “mathematics resilience” (9) indicates shortcomings in translating basic research findings into practice and in designing resilience development programs. In light of these findings, it can be said that the field of mathematics education psychology needs a more balanced and inclusive approach.

Research on affective factors in mathematics education between 1978 and 2025 reveals significant evolutionary patterns. A comprehensive review of 2,587 publications through thematic modeling and temporal analysis shows notable transformations in both the volume and thematic orientation of research focus. The findings presented below cover seven distinct thematic clusters identified using the Latent Dirichlet Allocation (LDA) method, as well as the developmental trajectories of these clusters over five-year periods and the structural relationships between research areas revealed by hierarchical clustering and multidimensional scaling analyses. The analysis reveals that teacher-focused research and math anxiety have emerged as dominant themes in recent years, while also identifying underdeveloped research areas that hold significant opportunities for future academic research.

Table 2 shows the development of articles published on affective factors in mathematics education between 1978 and 2025 and their distribution according to seven main topics.

**Table 2:** Distribution of articles by topic and publication period (1970–2025) based on topic modeling.

Topic number	1978–1983	1984–1988	1989–1993	1994–1998	1999–2003	2004–2008	2009–2013	2014–2019	2020–2025	Total
Topic 1	0	1	0	0	2	2	4	13	20	42
Topic 2	11	7	18	23	25	36	89	250	538	997
Topic 3	2	1	2	2	2	4	16	31	65	125
Topic 4	0	0	1	3	3	22	31	100	210	370
Topic 5	6	4	10	15	20	31	77	144	207	514
Topic 6	0	3	1	3	8	6	42	132	212	407
Topic 7	0	1	0	1	0	4	3	30	93	132
Total	19	17	32	47	60	105	262	700	1,345	2,587

Looking at the “Total” row of the table, it can be seen that studies related to the affective domain in mathematics education have been steadily and rapidly increasing; particularly in the recent period (2020–2025), with a total of 1,345 articles, approximately 52% of all articles have been published. This shows that the topic has become one of the most interesting areas in the academic community today.

There are significant differences in research intensity between topic headings across all time periods.

Most Intensively Studied Topics Topic 2 (Factors Affecting Affective Factors—997 articles), Topic 5 (The Effect of Affective Factors on Achievement—514 articles), and Topic 6 (Anxiety in Mathematics Education and Instruction—407 articles). This intensity shows that researchers have focused primarily on investigating what affects affective variables, how they relate to success, and the problem of anxiety.

The least studied topic is Topic 1 (Technology and Materials in Studies Related to Affective Factors in Mathematics Education—Total: 42 articles). This low number of articles is closely related to the unique focus of the topic. Topic 1 brings together experimental and situational studies that focus on the formation and change of students’ affective characteristics and examine the application methods, effects, and effectiveness of digital technologies, learning materials, and pedagogical tools in this process. The main focus of these studies is technology; it involves technology in both the development and data collection and analysis stages. Due to this specific focus, it is logical that the number of articles in Topic 1 is lower than in other topics because technology is generally associated more with cognitive abilities and achievement, and pure affective skills are not the primary focus of technology researchers. Therefore, the number of rigorous, technology-centered studies focusing on affective outcomes in this area is still limited.

**Figure 4:**
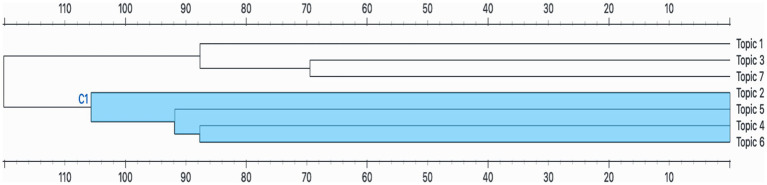
Hierarchical clustering findings through topic modeling.

According to the findings of the hierarchical clustering analysis (Figure 4), the topics examined were grouped into two main clusters: Cluster C1 (Topics 2, 4, 5, and 6) and Cluster 2 (Topics 1, 3, and 7). The order of convergence of the main C1 cluster reflects the fundamental research focus in the affective domain. The early and high similarity convergence of Topic 4 (Teachers and Teaching) and Topic 6 (Mathematics Anxiety), which form the basis of the cluster, indicates that the majority of the studies focused on the direct relationship between the anxiety phenomenon and the pedagogical role of teachers. The subsequent inclusion of Topic 5 (Student Achievement) in this relationship reinforces the significance of treating student achievement as the primary outcome variable in these studies. Topic 2 (Factors Affecting Affective Factors), the last component of the cluster, forms the most distant connection because it examines general variables affecting affective factors; this shows that Topic 2 focuses on variables that predict affective outcomes, unlike the other topics. The C1 cluster generally examines the central role of affective state in mathematics education within the educational and teaching processes and, in this respect, focuses directly on the pedagogical context.

The second cluster does not show as high a degree of similarity as C1, but it does exhibit a meaningful relationship within itself. In this cluster, Topic 3 (Demographic Variables) and Topic 7 (Family Influence) are primarily combined. The proximity of these two topics stems from their examination of the strong relationship between affective outcomes and the individual background (demographic variables) and the closest environmental factor, family influence. Topic 1 (Technology and Materials), on the other hand, is positioned further away from the other topics due to its focus on technology use, which can be included up to the analysis stage of the studies. The common point of this cluster is that it examines the contextual relationship of these three factors with affective factors in the educational environment, rather than focusing directly on education and teaching processes.

**Figure 5:**
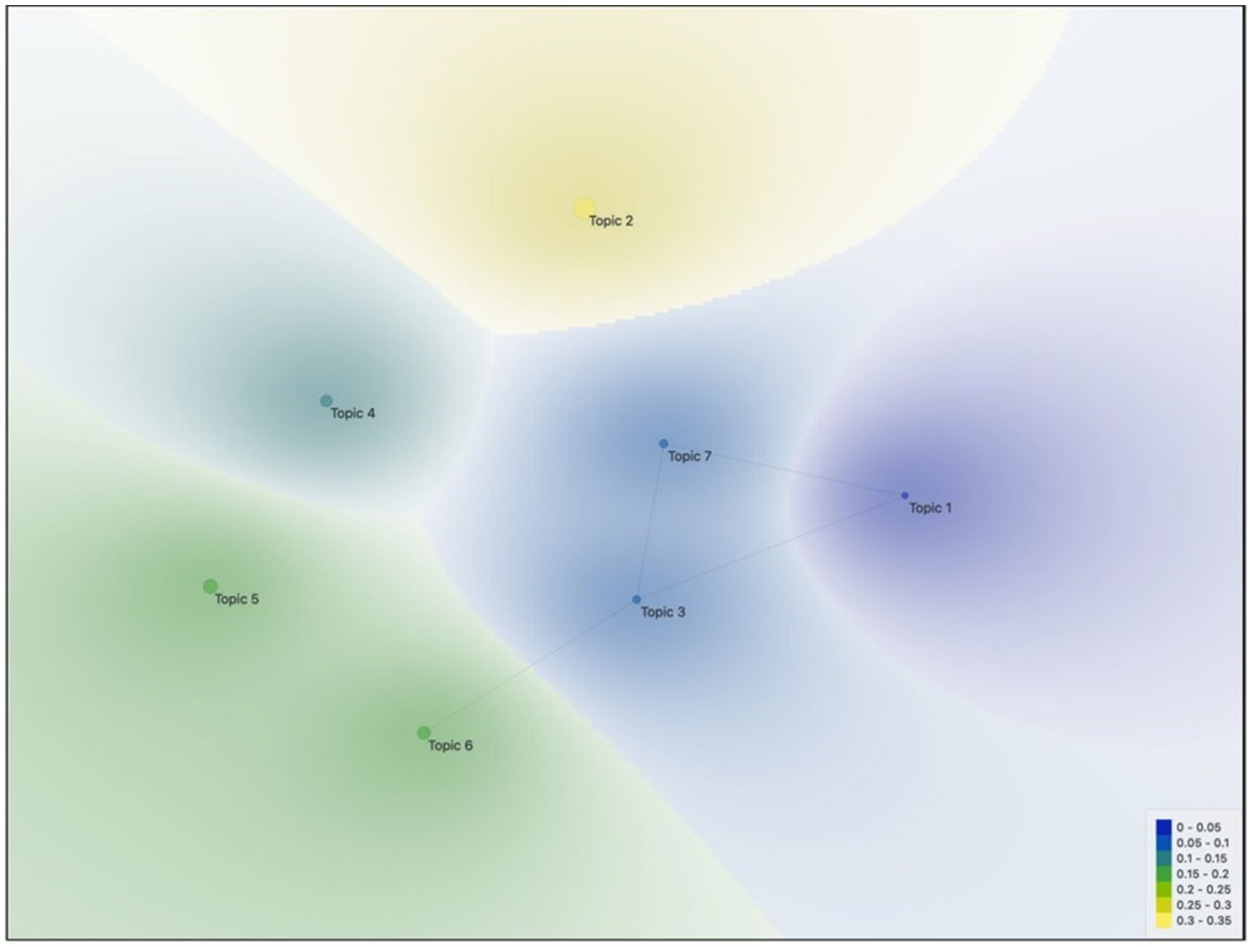
Multidimensional scaling of topics as a result of topic modeling.

Multidimensional scaling (MDS) analysis (Figure 5) visualizes the relationships between topics by positioning them on a two-dimensional plane through color transitions. Similarly, hierarchical clustering results also support the structure observed in the MDS map. In the graph, Topic 6, Topic 5, Topic 4, and Topic 2 are located close to each other and share a similar color spectrum. These topics together form cluster C1, representing studies focusing on the impact of teaching, learning, and success on affective factors.

In contrast, Topic 1, Topic 3, and Topic 7 form a distinct cluster structure. Topic 3 and Topic 7, in particular, are closely positioned and include studies related to family and demographic variables. Topic 1, on the other hand, is located further away from all other topics and represents studies focusing on the use of technology in mathematics education in different contexts.

When hierarchical clustering and multidimensional scaling (MDS) results are evaluated together, it is seen that studies related to affective factors in mathematics education are grouped around two main thematic axes. The first axis (Topics 2, 4, 5, 6) covers affective variables related to teaching, learning, and achievement, while the second axis (Topics 1, 3, 7) highlights contextual factors such as technology, demographic characteristics, and family. The spatial differentiation observed in the MDS map confirms the thematic clustering identified in the dendrogram analysis.

**Figure 6:**
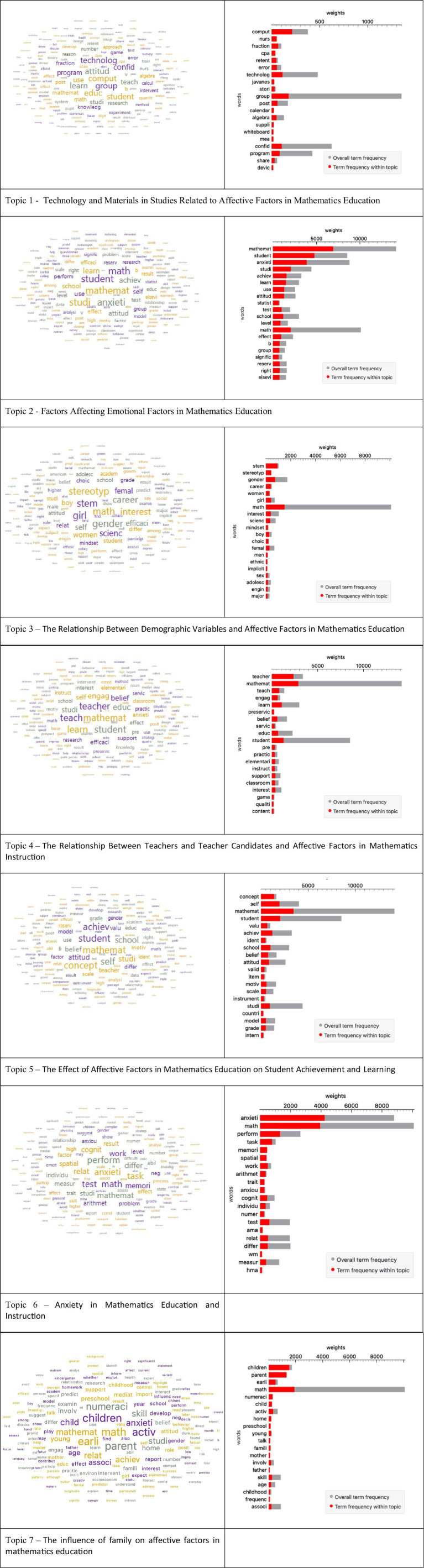
Word clouds of topics and LDAvis scales.

The word clouds and weight graphs (Figure 6) visualized with the keywords obtained as a result of topic modeling (Table 2) confirm the thematic focus of the seven identified topics.

Topic 1: Technology and Materials in Studies Related to Affective Factors in Mathematics Education clearly focuses on technology integration, as indicated by the keyword “computer” and the inclusion of various technological tools such as “device,” “calendar,” and “whiteboard” in the weight graphs (Figure 5). However, the fact that the keywords “teach” and “student” appear at the end of the list suggests that technology itself is the primary focus of these studies, with teaching or learning outcomes positioned as secondary objectives (Table 3).

**Table 3:** Ranking of word groups according to topics.

Topic	Key words
Topic 1—Studies Related to Emotional Factors in Mathematics Education Technology and Materials	comput, group, attitud, educ, technolog, learn, student, confid, teach, use
Topic 2—Factors Affecting Affective Factors in Mathematics Education and Instruction	mathematics, student, anxiety, study, math, achievement, learning, use, attitude, school
Topic 3—The Relationship Between Demographic Variables and Affective Factors in Mathematics Education	math, STEM, gender, stereotype, girl, career, interest, science, self, women
Topic 4—The Relationship Between Teachers and Teacher Candidates and Affective Factors in Mathematics Instruction	mathematics, teacher, student, learn, teach, education, engagement, belief, study, self
Topic 5—The effect of affective factors in mathematics education on student achievement and learning	mathematics, student, self, concept, achievement, school, study, attitude, belief, teacher
Topic 6—Anxiety in mathematics education and teaching	anxiety, math, perform, task, test, mathematics, relate, work, differ, cognitive
Topic 7—The influence of family on affective factors in mathematics education	math, children, parent, early, activity, numeracy, mathematics, anxiety, skill, relation

Topic 2: Factors Affecting Affective Factors in Mathematics Education includes words that point to teaching and learning outcomes, such as “student,” “school,” “learn,” “teach,” and “achieve,” along with different affective factors (“attitude,” “anxiety,” “self”) related to different affective factors. This combination reveals that the basis of the topic is to examine the complex interaction of affective variables on learning and teaching processes.

Topic 3: The Relationship of Demographic Variables to Affective Factors in Mathematics Education includes words related to social identity and structure, such as “gender,” “women,” “ethnic,” and “stereotype.” This shows that it examines the relationship between affective factors and social status and demographic characteristics. Furthermore, it is noteworthy that affective components such as “interest” and “mindset” are at the forefront in this topic, rather than general anxiety.

Topic 4: The relationship between teachers and teacher candidates and affective factors in mathematics education is characterized by the words “teacher” and “preservice” being the most dominant words in both the word cloud and weight graphs. While words indicating pedagogical action, such as “instruction,” are prominent, “belief” is dominant as an affective component. This shows that the studies primarily focus on teachers’ and teacher candidates’ beliefs and perceptions of self-efficacy regarding teaching.

Topic 5: The title “The Effect of Affective Factors in Mathematics Education on Student Achievement and Learning” indicates that the focus is on outcomes for students, as it includes the keywords “student” and “achieve.” Cognitive emotions such as “self-concept,” “attitude,” and “belief” are prominent in this regard.

Topic 6: Anxiety in Mathematics Education-Teaching, as the name suggests, includes “anxiety” as the fundamental affective factor, indicating that this emotion forms the main axis of related studies. Anxiety is examined based on its relationship with different cognitive and behavioral elements.

Finally, Topic 7: The family’s influence on affective factors in mathematics education, with the words “children” and “early” along with the word “parent,” suggests a focus on the effect of family relationships on affective factors, particularly in the preschool and early childhood periods. Anxiety is also seen to be a prominent factor in this regard.

**Figure 7:**
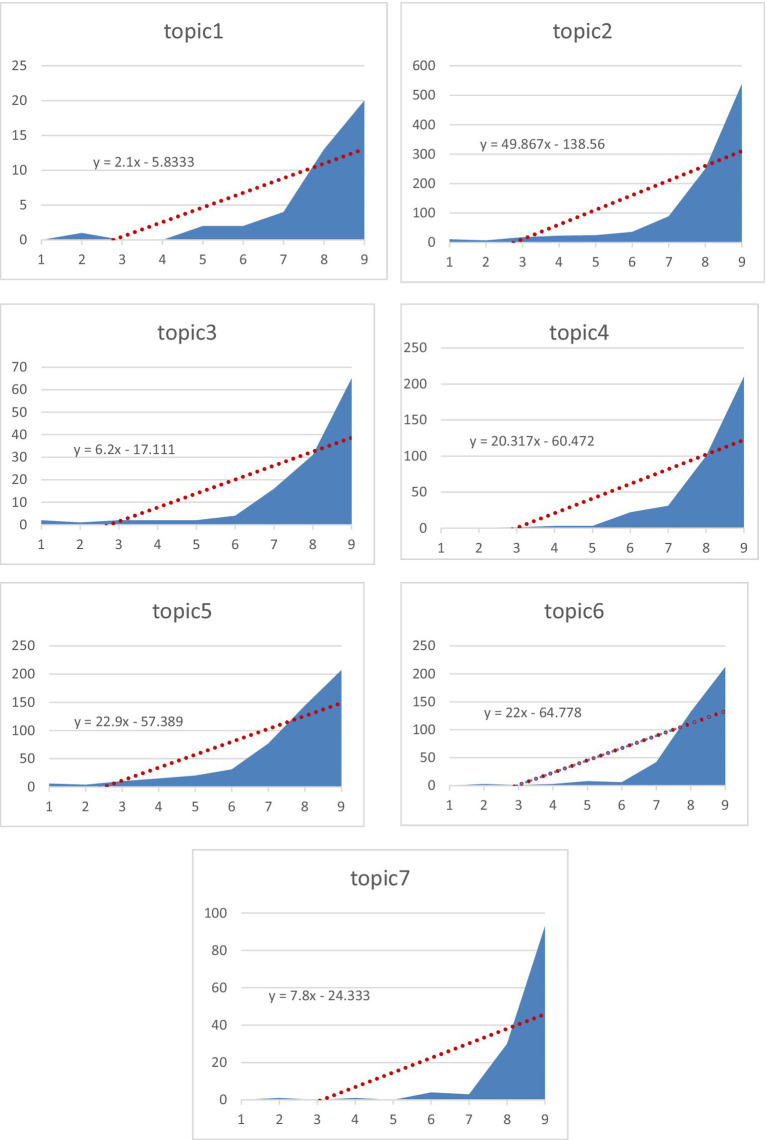
Volume and slope graphs of the topics in the field of affect/emotion in mathematics education.

An examination of the graphs (Figure 7) clearly shows that academic interest in affective factors in mathematics education has increased steadily and rapidly from 1978 to 2025. Although the growth trends for all topics are positive, this growth rate and focus of interest show significant differences over time. The turning point in the number of articles began in Topic 5 during the 2004–2008 period, while other mainstream topics (Topics 2, 3, 4, 6, 7) experienced their major leap starting in the 2009 period. These mainstream topics, namely factors affecting affective factors (Topic 2), predicting success (Topic 5), and math anxiety (Topic 6), have the highest growth rates and form the main body of the field. On the other hand, Topic 1 (Technology and Materials in Studies on Affective Factors in Mathematics Education) has the lowest growth rate (*m* = 2.1) compared to all other main topics. Topic 1’s low performance can be attributed to the central role of technology in its definition. This topic places technology at the center as both a tool and an analysis method for understanding and developing affective characteristics. However, since cognitive abilities are generally the primary focus of technology, technology-based studies focusing solely on affective skills have failed to attract the main interest of researchers working in the field of mathematics education, and interest has shifted to other topics. This situation suggests that technology-based studies directly related to mathematical affective skills have shifted their focus to technology experts outside of mathematics education or to cognition-focused research, and that interest in this specific area has relatively declined. On the other hand, Topic 4 (Teachers), with its increase after 2014, reveals that the importance of teachers’ own emotional states has grown exponentially in recent years.

**Figure 8:**
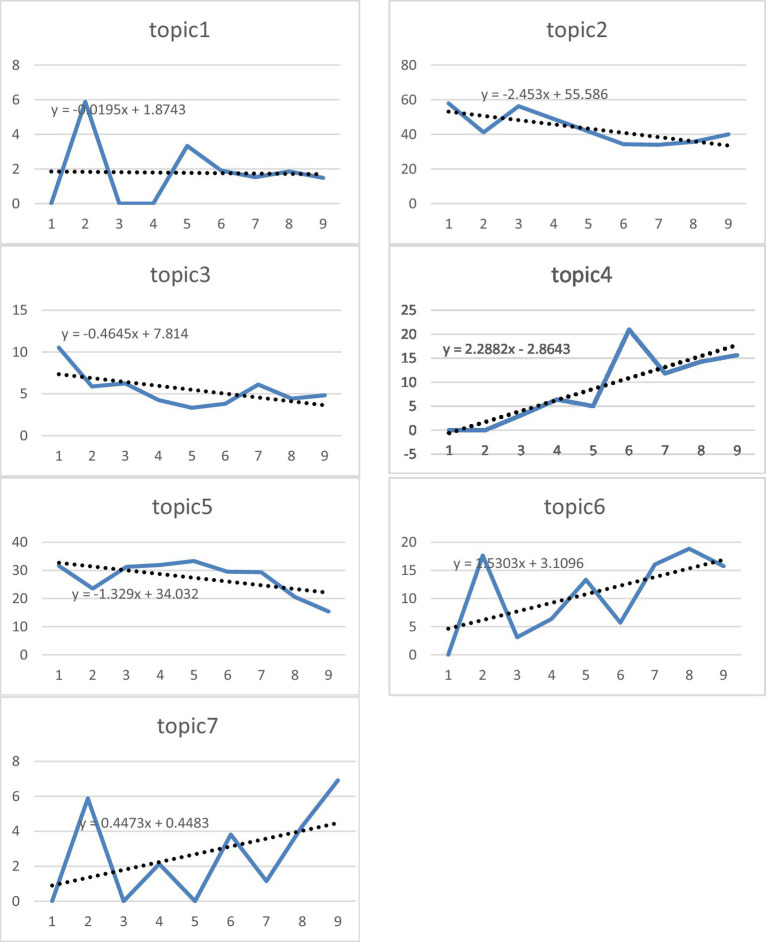
Percentage and acceleration graphs of topics in the field of emotion in mathematics education.

This analysis examines the relative percentage of each topic within the total publication volume, based on the periodic distribution of articles published between 1978 and 2025 (Figure 8). This reveals the evolution of academic interest in emotional factors in mathematics education over time. In the early period (1978–1993), Topic 2 (Factors Affecting Emotional Factors) and Topic 5 (The Effect of Success) were clearly dominant among the topics that formed the basis of the field. These two topics have long been prominent in the literature because they focus on both basic psychological processes () and the affective aspects of performance-based learning outcomes. However, the negative slope coefficients (*m*₂ = −2.453; *m*₅ = −1.329) indicate that the relative shares of these topics have decreased over time. This decrease is more indicative of research saturation in these topics and a shift in researchers’ interests toward more specific subfields than a decline in interest in the field. In contrast, Topic 4 (Teachers/Teacher Candidates) shows a remarkable increase over time. This area, which stands out as the topic with the highest positive slope with a value of *m*₄ = +2.2882, has shown strong growth, particularly since the 2000s, focusing on the effects of teachers’ beliefs, attitudes, and anxiety levels on student achievement and motivation. This indicates a shift in the focus of affective research in mathematics education from a student-centered structure to a teacher-centered structure. Similarly, Topic 6 (Mathematics Anxiety) also shows a positive slope with a value of *m*₆ = +0.5303, exhibiting a rapid rise, especially in the post-2010 period (2014–2025). This trend indicates that math anxiety has become a central problem area for both students and teachers, and that awareness of this issue has increased significantly in the academic community. On the other hand, Topic 1 (Technology and Materials) shows an almost stagnant trend (*m*₁ = +0.0195), failing to increase its relative interest over the period from 1978 to 2025. Although the absolute number of articles has increased, this increase has been parallel to the overall growth in publications. Therefore, technology-focused affective research has shown development proportional to the overall momentum of the field, but not exceeding it. This situation suggests that the association of technology with affective factors remains an underdeveloped subfield. In conclusion, throughout the period 1978–2025, technology-based and success-oriented studies in mathematics education research on affective factors have reached a saturation point, while teacher-themed and anxiety-oriented research orientations have rapidly increased. This picture shows that the field is evolving into a broader and more multidimensional structure by turning to new emotional dimensions and actors in its maturation process.

**Figure 9:**
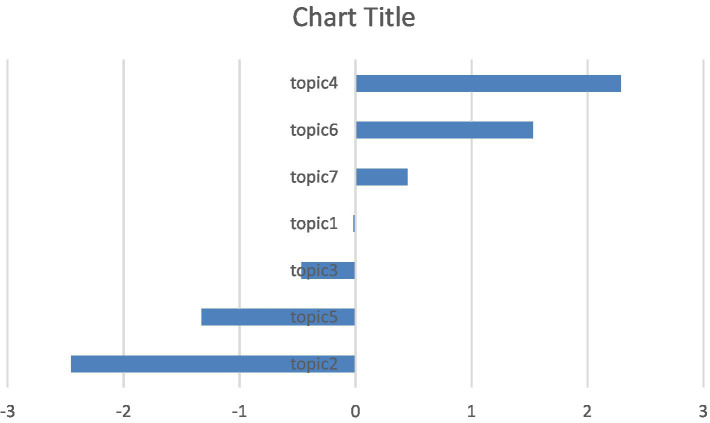
Comparison of acceleration values of emotion in mathematics education.

This bar chart (Figure 9) summarizes the relative interest dynamics (topic slope coefficients) of research on affective factors in mathematics education literature between 1978 and 2025. The graph clearly shows that the academic focus in the field has shifted over time from student outcomes to teaching actors and specific problem-solving.

The most pronounced upward trend is observed in Topic 4 (Teachers/Teacher Candidates) and Topic 6 (Mathematics Anxiety). Topic 4 having the highest positive slope indicates that affective research is placing greater emphasis on the role of the teacher and that interest in the field is shifting toward the impact of teaching factors on affective skills, as well as student performance. This situation reveals that educators’ affective competencies (belief, self-efficacy, anxiety) are accepted as a central variable for the quality of the learning environment and student motivation. The rise in Topic 6 confirms that math anxiety is increasingly recognized as a fundamental and priority issue in the academic community and that there is a growing need for solution-oriented work in this area.

In contrast, Topic 2 (Factors Affecting Affective Factors) and Topic 5 (The Effect of Success), which were dominant research areas in the early periods, have the largest negative slope values. This negative trend indicates that, despite the absolute increase in the number of articles, research saturation has been reached in these fundamental topics and *relative* interest in the field has declined. Consequently, these topics are now struggling to attract the primary interest of researchers, indicating that resources and interest are shifting toward more specific and application-centered emerging topics (Topic 4, 6). Other topics (Topics 1, 3, 7) follow a more stable trajectory, showing growth below or in line with the overall growth in the field.

## 4 Discussion

The findings of this study reveal that the academic research agenda on affective factors in mathematics education has undergone significant evolution from 1978 to the present day, and that this evolution is consistent with broader paradigmatic shifts in the field. The most striking finding is that, despite steady and exponential growth in the absolute number of articles, there has been a marked shift in the thematic focus of relative academic interest. The decline in the relative share of broad and fundamental psycho-educational topics, which were dominant in the early period, suggests that a kind of saturation has been reached in these areas. This situation can be interpreted as an indicator of the field’s maturation; researchers are now turning to more specific and contextual questions such as “in whom, how, and depending on what are these factors shaped?” rather than “are affective factors important?” (Leder and Forgasz, 2018; Grootenboer et al., 2015). Recent bibliometric reviews confirm that mathematics education research has increasingly shifted toward context-sensitive and mechanism-oriented questions, reflecting a more advanced stage of disciplinary development (Donthu et al., 2021). This transition signals a new phase in mathematics education research that could be called the third wave (Gomez, 2017).

The present study contributes uniquely to this literature by empirically demonstrating this paradigmatic transition through long-term trend topic analysis, offering longitudinal evidence that complements and extends earlier cross-sectional or short-period bibliometric studies.

The strongest evidence for this trend is the rapid increase in studies focusing on the affective factors of teachers and teacher candidates, indicating a systematic shift in mathematics education research from student-centered to teacher-centered perspectives. The literature consistently shows that teachers’ mathematical beliefs, anxieties, and self-efficacy shape not only their instructional practices but also students’ affective profiles, classroom experiences, and academic achievement (Peters, 2022). In particular, teachers’ mathematics anxiety may be transmitted to students through classroom interactions and emotional climate (Bekdemir, 2010; Beilock et al., 2010). Recent large-scale studies further demonstrate that teacher affect functions as a mediating variable between instructional quality and students’ emotional engagement (Zhao, 2024).

From a practical standpoint, this finding highlights teacher affect as a critical leverage point for intervention, suggesting that professional development programs targeting teachers’ affective competencies may yield indirect but substantial benefits for student engagement and achievement.

Recent research highlights the potential to further enrich this line of inquiry by incorporating growth mindset and computational thinking. Teachers’ growth mindset beliefs influence how mistakes, effort, and persistence are framed in the classroom (Dweck, 2006), while orientations toward computational thinking shape how problem-solving and digital tools are integrated (Wing, 2006; Weintrop et al., 2016). Emerging studies indicate that embedding these constructs jointly in teacher education reduces student anxiety and strengthens mathematical agency (Looi, 2024). However, the limited number of such integrative studies points to a clear research gap: the need for theoretically grounded models that explicitly connect teacher affect, instructional cognition, and student emotional outcomes.

Similarly, the increased focus on math anxiety reflects its reconceptualization as a multidimensional phenomenon intersecting with neuroscience and educational policy. Neurobiological evidence demonstrates its negative effects on working memory (Ramirez et al., 2018), reinforcing the urgency of evidence-based interventions (Dowker et al., 2016; Carey et al., 2016). Recent interdisciplinary studies integrate neurocognitive and affective data to design targeted interventions (Yu, 2023). Despite this progress, intervention research remains unevenly distributed across educational levels and cultural contexts, revealing another important gap for future inquiry.

On the other hand, studies addressing technology and instructional materials occupy a relatively limited position within the affective research agenda. While this may appear counterintuitive given digital transformation in education, it reflects the historical prioritization of cognitive outcomes in technology research (Hwang et al., 2015; Aliyu et al., 2021). Recent reviews emphasize that affective outcomes of educational technologies remain conceptually underdeveloped and methodologically challenging, especially in AI-supported environments (Gqoli and Baidoo, 2025). This finding should be interpreted cautiously, not as a lack of relevance, but as evidence of an underexplored and methodologically demanding research frontier. Well-theorized empirical studies are needed to clarify the conditions under which AI-supported environments reduce math anxiety and foster positive mathematical identities (Elsayed and Abdo, 2023).

Finally, the cluster centered on demographic variables and family influence underscores the sociocultural embeddedness of affective development. Mechanisms such as stereotype threat (Steele, 1997; Spencer et al., 2016) and intergenerational transmission of math anxiety (Maloney et al., 2015; Sweeney and Wilson, 2023) operate differently across contexts (El Chaabi and Younes, 2025). These results highlight a persistent gap in culturally responsive and equity-oriented affective research, particularly in underrepresented regions and populations.

This asymmetric distribution in the research agenda further reveals conceptual blind spots. Positive psychological constructs (enjoyment, curiosity, flow) and social factors (sense of belonging) remain underrepresented, limiting the explanatory power of dominant models (Pekrun and Linnenbrink-Garcia, 2024). Likewise, epistemological beliefs and metacognition are insufficiently integrated, despite their central role in linking cognition and affect (Hofer and Pintrich, 1997; Muis and Franco, 2009). The lack of integrated affective–metacognitive intervention studies represents a critical disconnect between theory and classroom practice.

## 5 Conclusion and recommendation

This study examined research trends in affective factors in mathematics education between 1978 and 2025 using trend topic analysis and revealed a dynamic but asymmetrical evolution of the field. The systematic shift from general themes to specific and contextual topics reflects increasing conceptual sophistication, yet the dominance of deficit-oriented constructs (particularly mathematics anxiety) indicates a problem-centered research paradigm.

In contrast, the marginal presence of positive psychology constructs such as enjoyment, curiosity, and flow highlights a significant imbalance. Although thematic clustering reveals meaningful structural patterns, the low representation of social and metacognitive factors indicates that the field remains conceptually fragmented rather than theoretically integrated.

The unique contribution of this study lies in its long-term, data-driven mapping of these imbalances, providing empirical evidence of not only what the field has prioritized, but also what it has systematically overlooked over nearly five decades.

To advance the field, future research must rebalance thematic priorities by expanding work on positive affective experiences and developing comprehensive frameworks that integrate affective, cognitive, metacognitive, and social dimensions. Qualitative and mixed-method studies are particularly needed to capture the lived emotional experiences of learners and teachers.

Experimental research at the intersection of technology and affective factors should be strengthened through theoretically grounded designs. AI-based adaptive systems, augmented reality, and immersive environments hold promise, but their affective impact must be tested rigorously and cautiously.

From a policy and practice perspective, the findings call for the explicit integration of affective goals into teacher education and curriculum design. Supporting teachers’ affective competencies, embedding affective objectives in curricula, and implementing early screening mechanisms for math anxiety can translate research insights into sustainable educational change.

Finally, while this study offers a comprehensive longitudinal overview, its reliance on trend topic analysis limits insights into intellectual structures and collaboration patterns. Future bibliometric studies integrating co-citation, bibliographic coupling, and keyword co-occurrence analyses can extend this work by revealing theoretical lineages and emerging research fronts. Such multi-method approaches will enhance the strategic development of a more balanced, inclusive, and impactful affective research agenda in mathematics education.

## Data Availability

The original contributions presented in the study are included in the article/supplementary material, further inquiries can be directed to the corresponding author.
